# Creating an AFU Learning Community Group to Combat Ageism

**DOI:** 10.1093/geroni/igaa057.1729

**Published:** 2020-12-16

**Authors:** Carrie Andreoletti, Andrea June

**Affiliations:** Central Connecticut State University, New Britain, Connecticut, United States

## Abstract

Central Connecticut State University, a regional public university, joined the AFU global network in 2017. Through efforts to teach people what it means to be an AFU and infuse the AFU principles and practices into our campus culture, we recognized that our work must also include educating our faculty colleagues, staff, and administration about ageism and aging in general. Toward this end, we utilized our Center for Teaching and Faculty Development’s small grant program to develop an AFU learning community group (LCG) that includes faculty, staff, and older learners interested in the AFU movement and how they can contribute to the AFU effort on our campus. This presentation will discuss how we have used the LCG as an opportunity to combat ageism and increase aging literacy by sharing resources such as Ageism First Aid, Reframing Aging, and AARP’s Disrupting Aging classroom. Part of a symposium sponsored by Age-Friendly University (AFU) Interest Group.

